# The Trustees Can’t See It

**Published:** 1908-11

**Authors:** 


### THE TRUSTEES CAN'T SEE IT.

The State Boad of Medical Examiners having been told by Assistant Attorney General Sluder that the Board must pay the costs of enforcing the law against quacks (see leading editorial in this issue), suggested that the State Medical Associations furnish the money, as the Board gets in scarcely enough to pay expenses of doing business. The Trustees of the Association held a meeting, at which the Secretary and Editor of the State Association journal was present, and the proposal was laid before them. I am indebted to Dr. Chase, said Editor and Secretary, for advance sheets of his editorial on the subject (November number), which I reproduce below. It will be seen by comparing their decision with my own views on the subject, in leading editorial in this issue, that they coincide exactly. It is the duty of the State to enforce its laws; and to talk of the Examing Board paying for the enforcement of this law—which is in the interest of the whole people—is as absurd as to expect the Quarantine Department to pay for enforcing quarantine. Such course would put the medical profession in the aspect of persecutors and not prosecutors.

"ATTITUDE OF ASSOCIATION TRUSTEES.

"In such a crisis, the Board of Medical Examiners turns to the people for help, and especially to the medical profession, which best understands the need of public health protection. The Board has made no official appeal to the State Medical Associations, but the representatives of the various schools on the Board have appealed in person to their respective association officers. Our Trustees were asked for a thousand dollars to employ counsel for the Board. They have given the subject earnest consideration, and, while they recognized the importance and necessity of help for the Board, they have refused to make the appropriation requested. The grounds seem well taken. They believe this is not a fight of legalized doctors in the associations against unlegalized doctors outside of the associations. It is the fight of the whole people for self-protection. The medical profession led the people to pass their own laws through their own Legislature. A law was put on the statute books whereby every county could protect itself from unqualified medical attendants. It is every county's duty to enforce all its own laws. Before the higher courts, the Attorney General's aid should be invoked. If more aid is needed, the medical profession should only lead the people in protecting their law as it did in helping them to obtain their law. The Board is not a creation of the medical profession, but is appointed by the Governor and answerable to him and the people. The Board never refers matters to the medical associations. Medical associations are not responsible for wise or unwise actions of the Board, and should not be called upon for aid in justifying its acts. An attorney paid by medical associations to fight illegal doctors places the Board in a false light and jeopardizes the cause of the people by the appearance of medical persecution. It is believed that many physicians in all schools will not be willing to subscribe money for the general legal uses of the Medical Examining Board who would willingly give money to support the constitutionality of the law. Again, the Trustees believe that in so questionable and important a matter, the House of Delegates is alone sufficient to authorize such an appropriation, involving as it does a sum which is calculated to temporarily embarrass the work of the Association already undertaken."—*Advance sheets Texas State Journal of Medicine,* November, 1908.

The House of Delegates, which will not meet until next May, would make a grave mistake should they authorize the expenditure of its fund for this purpose.

